# The value of earlier-in-life systolic and diastolic blood pressure for cardiovascular risk prediction

**DOI:** 10.1016/j.isci.2024.109097

**Published:** 2024-02-02

**Authors:** Andreas Leiherer, Wolfgang Brozek, Axel Muendlein, Hanno Ulmer, Christoph H. Saely, Peter Fraunberger, Gabriele Nagel, Emanuel Zitt, Heinz Drexel, Hans Concin

**Affiliations:** 1 Vorarlberg Institute for Vascular Investigation and Treatment (VIVIT), Feldkirch, Austria; 2 Agency for Preventive and Social Medicine, Bregenz, Austria; 3 Institute of Medical Statistics and Informatics, Medical University of Innsbruck, Innsbruck, Austria; 4 Department of Internal Medicine I, Academic Teaching Hospital Feldkirch, Feldkirch, Austria; 5 Private University in the Principality of Liechtenstein, Triesen, Liechtenstein; 6 Institute of Epidemiology and Medical Biometry, Ulm University, Ulm, Germany; 7 Medical Central Laboratories, Feldkirch, Austria; 8 Department of Internal Medicine III, Academic Teaching Hospital Feldkirch, Feldkirch, Austria; 9 Landeskrankenhausbetriebsgesellschaft, Academic Teaching Hospital Feldkirch, Feldkirch, Austria; 10 Drexel University College of Medicine, Philadelphia, PA, USA

**Keywords:** Health sciences, Geriatrics, Public health, Biological sciences

## Abstract

Blood pressure (BP) varies over a lifetime. This cardiovascular observation study (OS) compared the predictive value of earlier- and later-in-life blood pressure (BP) in 1,497 cardiovascular disease patients utilizing readings taken during a health survey (HS) and 15 years later from the same subjects at the baseline of this OS. Prediction of the cardiovascular risk during the OS follow-up (21 years) was significantly more effective if the earlier BP readings at HS were used instead of recent OS readings (NRI = 0.30, p < 0.001). For HS readings, each 10 mm Hg increase of systolic and diastolic BP was associated with a 17% and 20% higher risk, respectively. At OS, systolic BP lost significance and diastolic BP reversed its association. Noteworthy, different BP categorizations (European vs. US guidelines) yielded similar results. This study highlights the poor predictive power of BP readings in elderly cardiovascular disease patients but emphasizes the significant prognostic value of earlier-in-life BP.

## Highlights

• Earlier vs. later-in-life BP readings of the same patients were compared
• Earlier readings are better cardiovascular risk predictors than later readings
• ESC/ESH and ACC/AHA categories of BP are comparably predicting cardiovascular risk
• Preserving early-in-life BP data ensures reliable risk prediction later in life

## Graphical abstract

**Figure:**
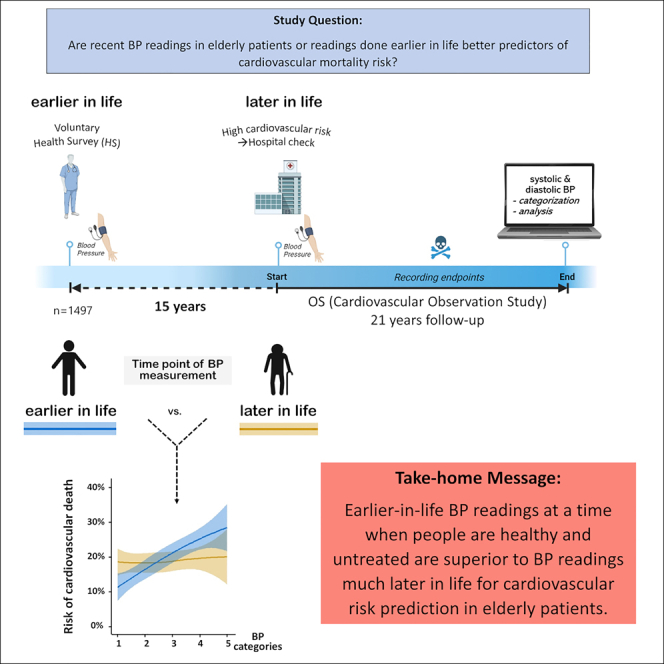

## Introduction

Blood pressure (BP) plays a pivotal role in cardiovascular disease (CVD) risk, particularly contributing to fatalities in older age groups.^1^ It is noteworthy that BP undergoes dynamic changes throughout an individual’s lifespan.^2^^,^^3^^,^^4^ These changes are intricately linked with the lifetime risk for CVD.^5^ Hence, the routine measurement and accurate recording of BP hold paramount importance in risk prediction, clinical practice, and public health interventions aimed at mitigating CVD.^1^^,^^2^^,^^3^^,^^4^^,^^5^ Nevertheless, the absolute risk increase for CVD with rising BP is relatively modest at lower BP levels, especially in younger age groups.^6^ The absolute annual differences in cardiovascular mortality with higher BP are much greater at older ages.^1^ On the other hand, while BP tends to rise with age, it often exhibits a decline toward the end of life.^7^ Additionally, the prevalence of diseases and the need for medical treatments escalate with advancing age, which confounds BP measurements in elderly patients. Consequently, the utility of BP measurements for predicting risk in the elderly has become a subject of questioning.^8^^,^^9^^,^^10^

Conversely, a former measurement when patients were younger and healthier may be less biased, but it is not clear if such early measurements are meaningful or even more valuable than recent measurements for risk prediction in the elderly.

Apart from that, comparing systolic and diastolic measurements, there is a focus on systolic BP regarding therapeutic decision-making.^11^ In contrast, the utility of diastolic BP is disputed,^10^^,^^12^ particularly in light of the U-shape of the association between diastolic BP and cardiovascular risk.^12^^,^^13^ Nevertheless, both, systolic and diastolic BP are used and combined in recent BP guidelines proposed by the US (American College of Cardiology [ACC]/American Heart Association [AHA]) and Europe (European Society of Cardiology [ESC]/European Society of Hypertension [ESH]),^14^^,^^15^ though they take a different position defining BP thresholds.

This opens two questions: First, to what extent are both measurements, systolic and diastolic BP, good predictors of disease, and if so, at which stage of life, e.g., in mid or late adulthood? Second, has the application of different BP categorization thresholds, ACC/AHA versus ESC/ESH, a significant impact on risk prediction results?

In the present investigation, we had the unique opportunity to combine datasets of a cohort recruited to two studies taking place 15 years apart. Hence, we aimed to investigate whether recent systolic and diastolic BP readings or earlier readings, done 15 years before in a healthier state, are a better predictor of cardiovascular risk in elderly patients and whether BP categorization differences between the US and Europe may play a role in that.

## Results

### Patient characteristics

Characteristics of the included subjects at the health survey (HS) and the observation study (OS) baseline are summarized in Table 1. At the OS baseline, subjects were about 15 years older (median 15.1 years, IQR 11.2–19.8 years) having a mean age of 65 years with 18% being 75 years or older. The distribution of systolic and diastolic BP between HS and OS (Figure S3) and the medians of systolic and diastolic BP at HS and OS appeared to be comparable. Overall, there was no striking shift from HS to OS (Figure S4). There was only a weak correlation between systolic BP at HS and systolic BP at OS (r = 0.223; p < 0.001) and between diastolic BP at HS and diastolic BP at OS (r = 0.118; p < 0.001, Figure S4). Systolic and diastolic BP were moderately correlated at HS (r = 0.656; p < 0.001) and at OS (r = 0.569; p < 0.001; Figure S5).

**Table 1:** Patient characteristics

	HS	OS	p-value
	N = 1497	N = 1497	
Age, mean years (median; IQR)	51 (51; 43–58)	65 (66; 58–73)	<0.001
Male sex, %	64	64	-
BMI, mean kg/m^2^ (median; IQR)	27 (26; 24–29)	27 (27; 25–30)	<0.001
Systolic blood pressure, mean mmHg (median; IQR)	139 (140; 125–150)	138 (140; 125–150)	0.096
Diastolic blood pressure, mean mmHg (median; IQR)	85 (80; 80–90)	81 (80; 75–90)	<0.001
Current smoking, %	36	18	<0.001

Characteristics of patients assessed at the health survey (HS) and 15 years later at the baseline of the cardiovascular observation study (OS). IQR denotes interquartile range.

The median follow-up duration was 12.7 years (IQR: 8.0–14.3). Out of the 1497 patients, 705 died from any cause during follow-up period (47%), and 284 (19%) experienced cardiovascular deaths.

### Association between BP categories and mortality during OS

**Figure 1:**
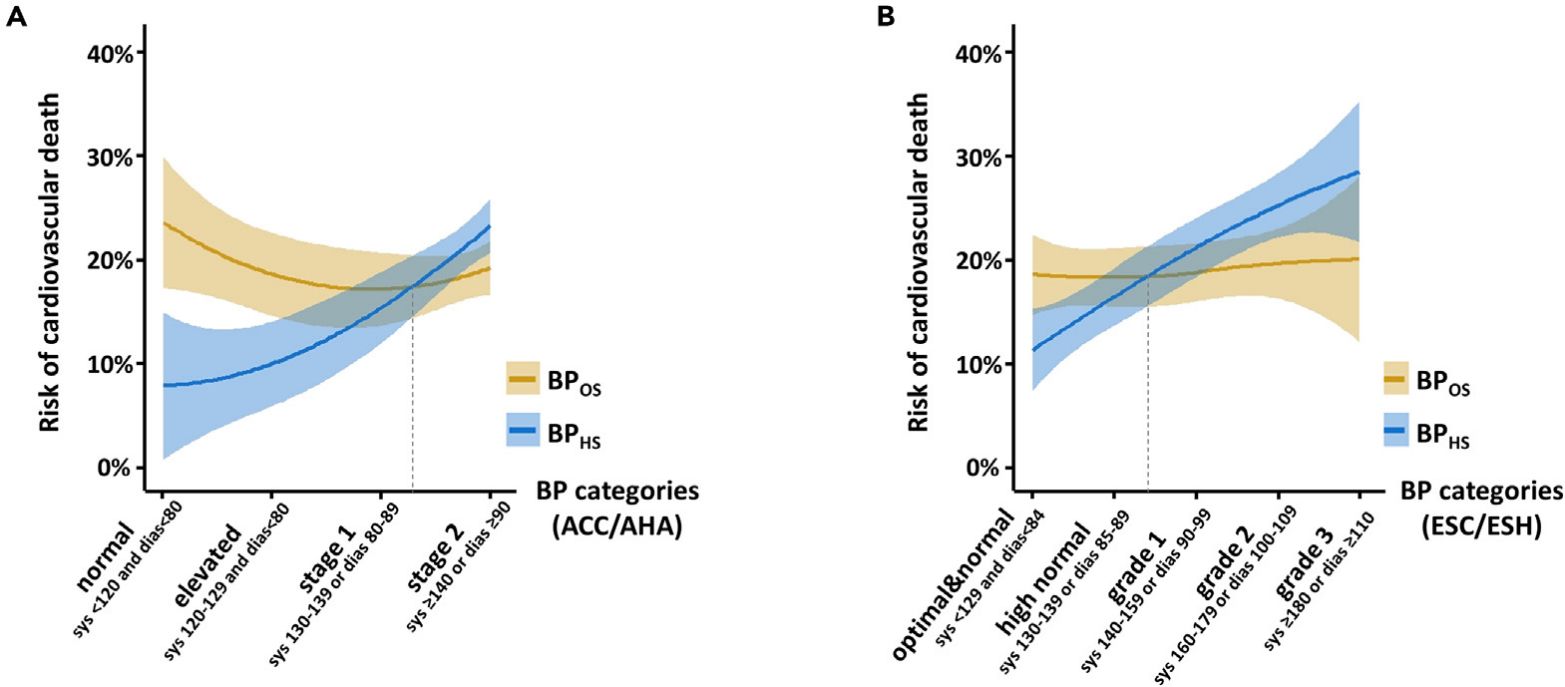
Risk curves for cardiovascular mortality with blood pressure categories measured at the health survey and the cardiovascular observation study (A and B) Curves are reflecting the risk of cardiovascular mortality during the follow-up of the cardiovascular observation study (OS) associated with blood pressure at OS or the health survey (HS). Curves are calculated according to loess (locally weighted scatterplot smoother) with 95% confidence intervals. BP categories were built using systolic and diastolic measurements according to ACC/AHA guidelines (A) and ESC/ESH guidelines (B), which are applying different cutoffs. The approximate crossing point between OS and HS-derived curves is indicated by a dashed line.

Figure 1 depicts the association between the BP categories, either according to ACC/AHA guideline thresholds or according to ESC/ESH guideline thresholds, and cardiovascular mortality during the OS using BP measurements done at the baseline of the OS (BP_OS_) or BP measurements done 15 years earlier at HS (BP_HS_). Applying the ACC/AHA categories demonstrates an approximately linear trend when using BP_HS_ for estimating cardiovascular mortality but a U-shaped and overall inverse relation when using BP_OS_. Comparable findings were made in terms of overall mortality (Figure S6). Although this discrepancy of risk curves for BP_HS_ vs. BP_OS_ appeared a bit weakened, when applying the ESC/ESH instead of the ACC/AHA categorization, the overall trend and the approximate crossing point between risk curves for BP_HS_ vs. BP_OS_ was comparable (Figure 1). Taken together, this distinctly demonstrates that rather the time point of BP measurement (HS vs. OS) than the way of categorization (ACC/AHA vs. ESC/ESH) impacts risk prediction.

**Figure 2:**
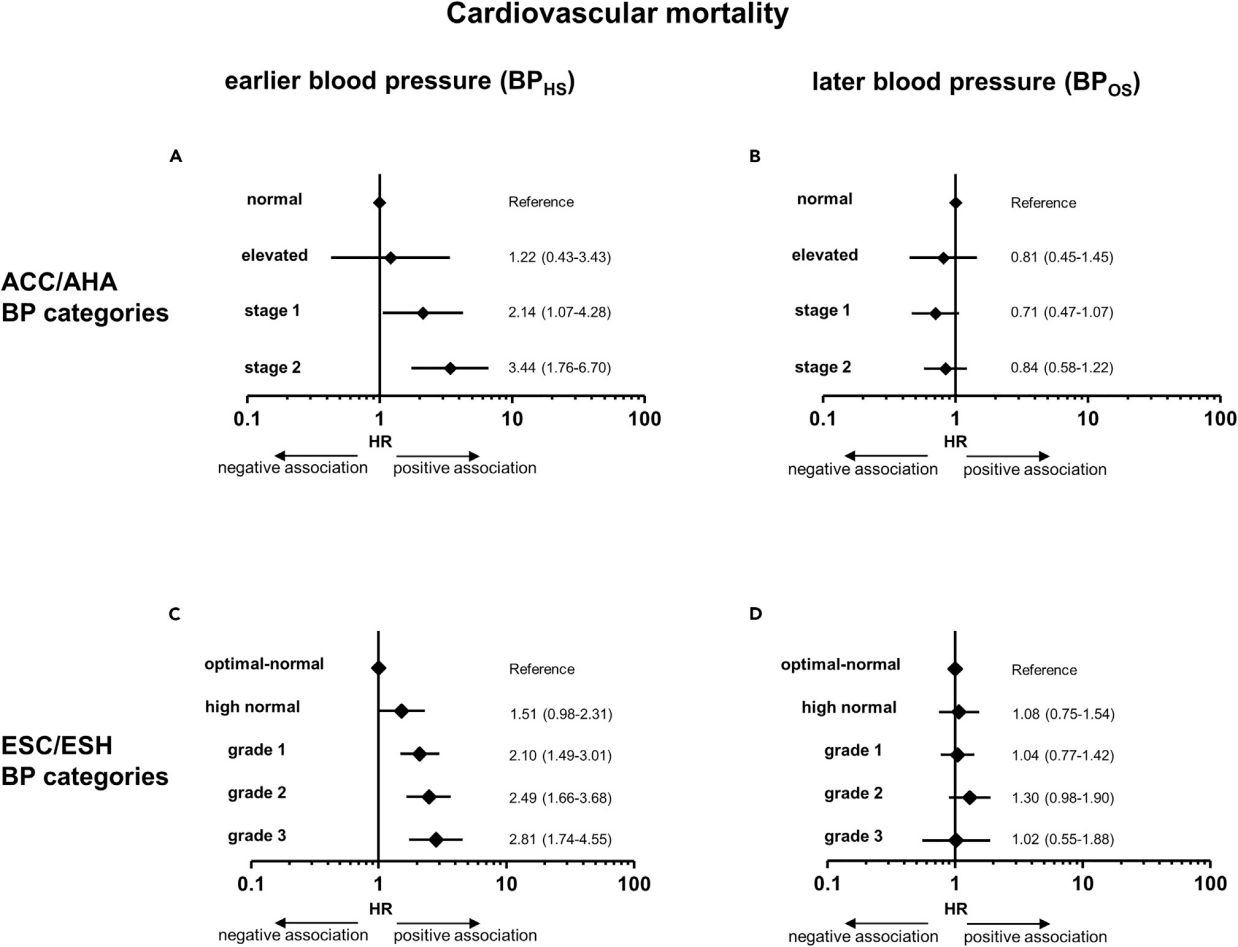
Association of blood pressure categories assessed at the health survey and at the baseline of the cardiovascular observation study with cardiovascular mortality (A–D) Forest plots represent hazard ratios with a 95% confidence interval of Cox regression analyses for the association between cardiovascular death and BP categories assessed at the health survey (HS; A + C) or the observation study (OS; B + D). Categories were built according to ACC/AHA (A + B) or ESC/ESH guidelines (C + D).

Applying a Cox regression analysis, the hazard ratio (HR) increases with increasing categories of BP_HS_ (Figure 2). Comparing the highest to the lowest BP_HS_ category, the HR for cardiovascular mortality was 3.44 [1.76–6.70] (p < 0.001) regarding ACC/AHA thresholds and 2.81 [1.74–4.55] (p < 0.001) regarding ESC/ESH thresholds. In contrast, no significant risk increase was observed when using BP_OS_ categories, neither with ACC/AHA nor with ESC/ESH thresholds (Figure 2). Regarding stepwise BP increase, a BP elevation measured at HS by one-step category significantly raised the mean cardiovascular mortality risk by 57% (1.57 [1.32–1.86], <0.001) when using the four-step ACC/AHA categorization and by 30% (1.30 [1.18–1.43], <0.001) when using the five-step ESC/ESH categorization. In contrast, when using OS readings, there was no significant risk increase (Table S1). Multivariate adjustment (including age, Δtime, sex, BMI, LDL-cholesterol, HDL-cholesterol, LP(a), anti-hypertensive medication, diabetes status, and smoking status) moderately attenuated but did not abrogate the significant association between cardiovascular mortality and BP. This was true with ACC/AHA as well as with ESC/ESH guideline thresholds (Table S1). Comparable findings were made for overall mortality (Figure S7). Multivariate adjustment had also no material impact on the association between BP at HS and the risk of overall mortality, but it abrogated this association when BP readings at OS were used (Table S1).

### Association between systolic and diastolic BP and mortality during OS

**Figure 3:**
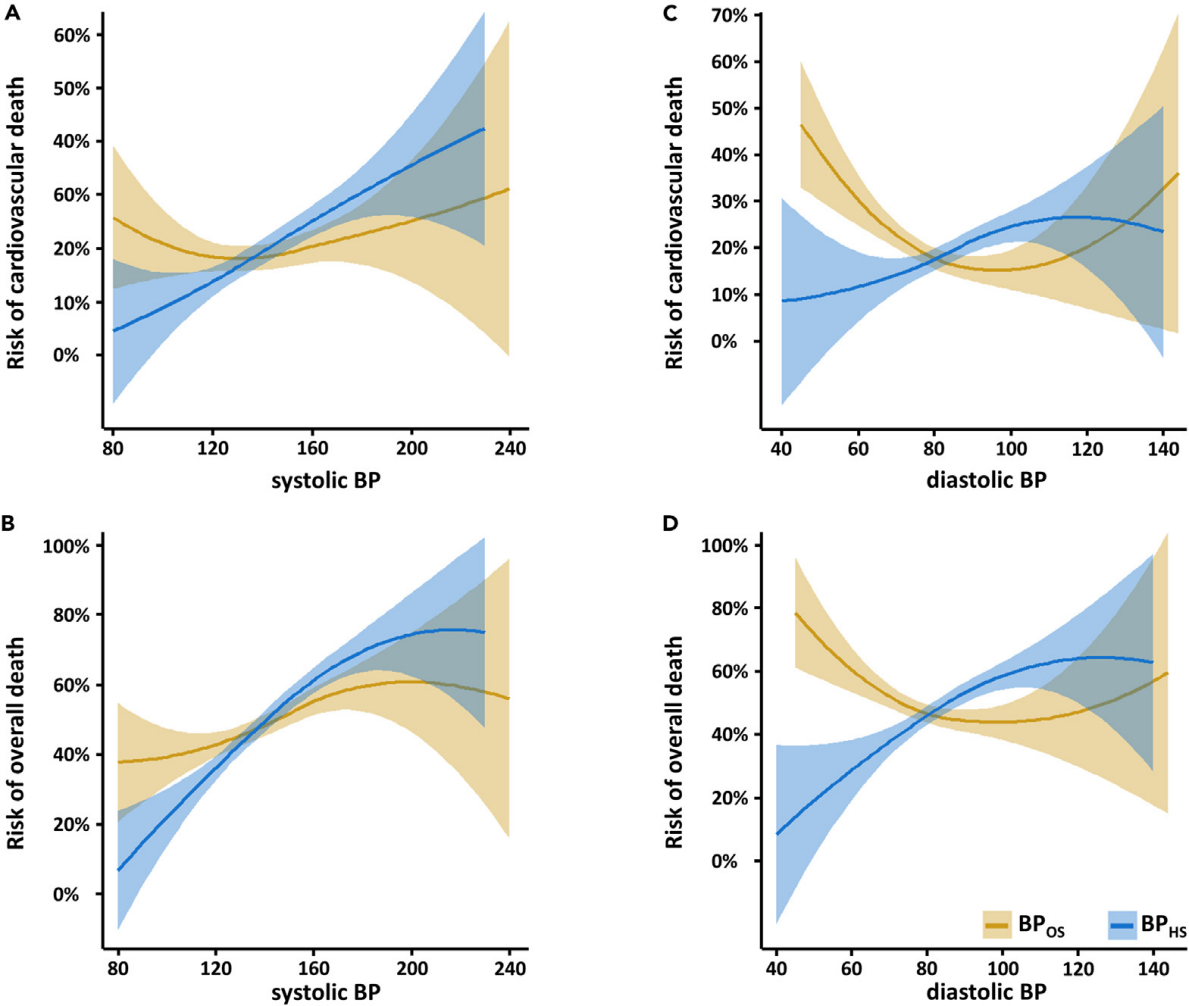
Risk curves for systolic and diastolic blood pressure measured at the health survey and at the cardiovascular observation study (A–D) Risk curves are calculated for BP at the HS and the baseline of the OS according to loess (locally weighted scatterplot smoother) fitting with 95% confidence intervals. It depicts the association of systolic BP with cardiovascular mortality (A), of systolic BP with overall mortality (B), of diastolic BP with cardiovascular mortality (C), and of diastolic BP with overall mortality (D).

Separate analyses for systolic and diastolic BP are depicted in Figure 3. Higher cardiovascular mortality in the OS was observed for higher systolic BP when measured at HS but not at the baseline of the OS. For overall mortality, a common trend was observed for higher risk with higher systolic BP measured both at HS and OS, though it was less pronounced for the latter. In the case of diastolic BP measured at OS, we found an inverse association with cardiovascular mortality as well as overall mortality. However, diastolic BP measured at HS was positively associated with cardiovascular and overall mortality.

Measured at HS, each rise in systolic BP by 10 mm Hg significantly increased the risk for cardiovascular mortality by 17% (HR = 1.17 [1.11–1.23], p < 0.001), but there was no significant increase when systolic BP was measured at OS (HR = 1.04 [0.98–1.102], p = 0.213). Using diastolic BP, we found an increase of 20% (HR = 1.20 [1.09–1.32], <0.001) with HS data, but an 18% decrease and consequently an inverse association with OS data (HR = 0.82 [0.73–0.92]). Comparable results were found for overall mortality (Table S2.)

### Association between change of BP and mortality risk

From HS to OS, a mean decrease of 1.1 mm Hg for systolic and 3.6 mm Hg for diastolic BP was observed. According to the AHA/ACC categorization, 28% of patients dropped into a lower BP category, 46% remained in the same category, and 26% entered a higher category. Applying the ESC/ESH categorization, this distribution was 38%, 29%, and 33%, respectively. In any case, dropping into lower BP categories from HS to OS was not associated with a reduction but an increase in cardiovascular or overall mortality risk: HR per one category lower was 1.24 [1.13–1.37] and 1.12 [1.05–1.19], respectively, for AHA/ACC and 1.16 [1.07–1.25] and 1.08 [1.03–1.14], respectively, for ESC/ESH.

Nevertheless, we also considered measurements at both time points for risk prediction. BP categories identified at HS were used for basic stratification and BP categories at OS for secondary stratification according to the changes in BP categories from HS to the OS baseline. Overall, the risk was mainly defined by BP at HS (Figure S8 and Table S3).

### Power of risk prediction

Irrespective of the way of categorization (ESC/ESH vs. ACC/AHA), BP at HS was a significantly better predictor of cardiovascular mortality than BP at OS (NRI_ESC/ESH_ = 0.29, p < 0.001; NRI_ACC/AHA_ = 0.30, p < 0.001). Separate analyses of systolic and diastolic BP showed that systolic BP at HS was the best predictor for cardiovascular mortality outperforming systolic BP at OS (NRI = 0.30, p < 0.001), but also diastolic BP at HS (NRI = 0.27, p < 0.001). Similar findings were obtained for overall mortality (Table S4).

## Discussion

### Main findings

We found that the varying categorization of systolic and diastolic BP measurements according to the ESC/ESH guidelines and the ACC/AHA guidelines had no material impact on risk prediction regarding cardiovascular mortality. However, we found that, irrespective of both categorization concepts, BP measured at HS was significantly more powerful in predicting cardiovascular and overall mortality than those measured 15 years later at OS. Systolic as well as diastolic BP measurements at HS were significantly positively associated with either overall or cardiovascular mortality. When we used measurements at OS instead, we found this association diminished or even abrogated for systolic values, and in the case of diastolic values, it actually became an inverse relation. Though some previous studies have investigated BP over several decades,^16^^,^^17^ this is, to the best of our knowledge, the first investigation that compared the predictive power of two readings of systolic and diastolic BP separated by such a long time, for cardiovascular and overall mortality in cardiovascular risk patients.

### Earlier-in-life BP readings vs. later-in-life readings

While we are aware of the confounding factors affecting BP measurements in elderly atherosclerotic cardiovascular disease (ASCVD) patients, especially in the case of diastolic BP, the predictive value of a possible alternative - BP measurements taken earlier in life when patients were younger, healthier, and untreated - has not yet been analyzed before this study.

In advanced age, cardiovascular risk prediction becomes a more significant concern compared to earlier stages of life.^1^^,^^18^ The present study, however, found that the aging of our patients from mid-life (50 years, at HS) to more advanced age (65 years, at OS) was linked with a significantly different BP categorization. Therefore, the prediction of overall and cardiovascular mortality risk changes as well, and it was significantly better when BP readings from mid-life were used instead of recent readings when patients were already at an advanced age.

The poor predictive power of BP in our OS was not surprising to us. It is already known that the prognostic significance of BP components clearly depends on age.^8^ Apart from age itself, further late-life characteristics including comorbidities and frailty impact blood pressure readings.^9^ Antihypertensive therapy is also an important confounder,^10^ lowering BP and largely reducing cardiovascular event risk and all-cause mortality in elderly patients,^19^ which explains the poor value of BP readings later in life for risk prediction.

Regarding the limitation of confounding effects, a previous study in individuals without hypertension or other traditional ASCVD risk factors has revealed a gradual increase in coronary artery calcium and ASCVD with increasing systolic blood BP beginning at 90 mm Hg.^20^ This aligns with the linear increase of cardiovascular risk with increasing systolic BP in our study at HS when the individuals were younger and in a healthy condition. Furthermore, recent Mendelian randomization studies also found a linear relationship between cardiovascular outcome and diastolic blood pressure.^13^^,^^21^ This is also in line with our data at HS because early-in-life readings are less biased than later-in-life readings and in that may approximate genetic variant testing, which excludes any bias by lifetime. However, with increasing age, systolic BP rises disproportionately relative to diastolic BP, which is the consequence of a pathologic reduction of arterial compliance by stiffening of the arterial wall.^22^ In our study, in which, at the OS, 18% of patients were 75 years and older, we found an ambiguous relation between diastolic BP and mortality risk: A U-shaped and inverse relation was seen for diastolic BP measured at OS but not at HS when the very same patients were younger and healthier. It is well known that around 50 to 60 years of age diastolic BP stops rising and starts falling.^2^^,^^3^^,^^16^ Concerning the coronary heart disease risk, a positive to negative shift with age for the relation with diastolic BP has already been found in the Framingham heart study.^23^ Similar findings are now reported by us regarding cardiovascular and overall mortality. The positive association between diastolic BP and mortality risk reversed when participants transitioned from mid-life (50 years, at HS) to advanced age (65 years, at OS).

### The value of systolic and diastolic BP readings

The value of diastolic BP is controversial.^24^^,^^25^ On the one hand, it has been claimed that particularly in middle-aged subjects diastolic BP is of better prognostic value than systolic BP^8^^,^^23^ and may provide additional prognostic utility beyond systolic BP.^26^ On the other hand, it has been repeatedly argued during the previous decades that for risk prediction and therapeutic decision-making, systolic BP is more informative than diastolic BP.^1^^,^^11^ In addition, many observational studies have reported a U-shape for the association of diastolic BP and cardiovascular risk^12^^,^^13^ and a more recent nationwide population-based study in Korea, Choi et al.^10^ have reported an inverse relationship between diastolic BP and cardiovascular events for measurements in men ≥55 years. Thus, concerns have been raised regarding the role of diastolic BP for the definition of hypertension and as a criterion in recent guidelines.^10^^,^^27^^,^^28^^,^^29^ In our study, we also found a U-shape association and an overall inverse relation between diastolic BP and cardiovascular risk – in case of readings at OS. However, when taken at HS, both BP readings, systolic and diastolic ones, were significant predictors of cardiovascular deaths as well as overall deaths. Diastolic BP only fails as a predictor if applying later readings at OS, when people were 15 years older.

### Added value of this study

This study is the first to compare the predictive power of two sets of systolic and diastolic BP readings taken with a significant 15-year time gap in identical subjects, focusing on both cardiovascular and overall mortality. The results indicate that when it comes to predicting mortality risk in elderly ASCVD patients, BP measurements obtained earlier in life were considerably more effective than the recent measurements which were taken 15 years later. Specifically, only systolic and diastolic BP readings from earlier in life demonstrated significant positive associations with cardiovascular and overall mortality, while recent systolic and diastolic BP measurements failed. Notably, these findings remained consistent, regardless of the variations in categorization of systolic and diastolic BP measurements between the ESC/ESH guidelines and the ACC/AHA guidelines.

These findings are important for two reasons. On the one hand, they support the concerns about the use of diastolic BP in elderly patients at elevated risk. On the other hand, even diastolic BP will give important information about future risk when measured at mid-life and in a healthy state. It only fails when measured at a more advanced age. We, therefore, partly agree with those who suggest that diastolic readings may be neglected or at least should be interpreted with caution, due to their biased association with cardiovascular risk. However, we underline that this accounts only for readings in elderly and biased patients with a high preexisting risk.

Moreover, our study further underlines previous conclusion that healthy blood pressure control plays an important role in preventing cardiovascular disease.^20^ Early readings, as shown here for BP or shown recently for total cholesterol readings,^30^ as well as early screening for prediabetes and diabetes^31^ are therefore not only valuable for risk prediction, but they also help achieve earlier treatment of patients at risk.

### Strengths and limitations of the study

One particular strength of our study lies in its design because it was conducted in a well-defined geographical area with low migration thus facilitating a high follow-up rate. Further, it comprised data from middle-aged participants and of the same participants a median time of 15 years later when they had become cardiovascular risk patients. This reflects a real-world situation.

A potential limitation is that our study participants were, at least at the time point of HS recruitment, healthy volunteers and might represent a particularly health-conscious Caucasian population. Subsequently, they were patients in the cardiology unit with a high cardiovascular risk. Therefore, this is a biased population that does not represent the general population. Furthermore, we compared the BP of patients only at two time points. More time points, including even earlier BP readings, would be necessary to determine the optimal timing or any cutoffs of measurement for risk prediction. We also did not analyze isolated systolic hypertension and summarized optimal and normal BP in the case of the ESC/ESH guidelines. Finally, there is no data on BP-lowering treatment in the HS. Moreover, as an established cardiovascular disease may affect BP, we cannot rule out reverse causality for the relation of BP at OS and risk prediction. That said, this again reflects clinical reality and underlines the value and importance of our data. In addition, we want to emphasize that risk prediction should not be confused with defining hypertension and controlling BP for which recent readings and not past readings are appropriate.

### Conclusion

Taken together, this study underlines the poor predictive power of BP readings in elderly patients. On the other hand, it demonstrates that earlier-in-life BP data, taken when individuals were younger, healthy, and untreated, hold significant prognostic value, even for elderly CVD patients. This applies to both systolic and diastolic BP measurements. Therefore, it is advisable to record and preserve these earlier-in-life BP data for risk prediction later in life, when patients have become elder.

## STAR★Methods

### Key resources table

**Table:** 

REAGENT or RESOURCE	SOURCE	IDENTIFIER
**Deposited data**		

Vorarlberg Health Monitoring & Prevention Program	Rapp et al.; Ulmer et al.	Ref.^30^, https://pubmed.ncbi.nlm.nih.gov/16234822/ Ref.^31^, https://pubmed.ncbi.nlm.nih.gov/12788300/
ACC/AHA high BP guidelines	American College of Cardiology (ACC) and American Heart Association (AHA)	Ref.^14^, https://www.jacc.org/doi/10.1016/j.jacc.2017.11.006
ESC/ESH guidelines	European Society of Cardiology (ESC) and European Society of Hypertension (ESH)	Ref.^15^, https://pubmed.ncbi.nlm.nih.gov/30165516/

**Software and algorithms**		

SPSS 28.0 for Windows	IBM, Chicago, IL, USA	https://www.ibm.com/products/spss-statistics
R statistical software v. 4.3.1	Foundation for Statistical Computing, Vienna, Austria; R CRAN	https://www.r-project.org
HMISC	R CRAN	https://cran.r-project.org/web/packages/Hmisc/index.html
G∗Power 3 software version 3.1.9.7 for Windows	Heinrich Heine University Düsseldorf, Germany	Ref.^32^, https://www.psychologie.hhu.de/arbeitsgruppen/allgemeine-psychologie-und-arbeitspsychologie/gpower
GraphPad Software	San Diego, California, USA	https://www.graphpad.com

### Resource availability

#### Lead contact

Further information and requests for resources should be directed to and will be fulfilled by the lead contact Andreas Leiherer (Vorarlberg Institute for Vascular Investigation and Treatment (VIVIT), Academic Teaching Hospital Feldkirch, Carinagasse 47, 6800 Feldkirch, Austria; E-mail address: vivit@lkhf.at).

#### Materials availability

This study did not generate new unique reagents.

#### Data and code availability

• The patient data reported in this study cannot be deposited in a public repository in order to preserve patient privacy and confidentiality.
• This study did not generate new original code
• Additional information required to reanalyze the data reported in this paper or reproduce the results is available from the corresponding author upon reasonable request.

### Experimental model and study participant details

This observational study comprises 1497 participants of Caucasian origin with a mean age of 65 years at the baseline of the cardiovascular observation study (OS), living in Vorarlberg, the westernmost province of Austria. At the baseline, the participants underwent coronary angiography and/or Doppler ultrasound sonography for the evaluation of atherosclerotic cardiovascular disease (ASCVD). Recruitment started in 1999, and patients were followed for up to 21 years.

A median period of 15 years before that, all 1497 cardiovascular risk patients had participated in a large health survey (HS), the Vorarlberg Health Monitoring & Prevention Program,^32^^,^^33^ that comprised over 185,000 adult residents of Vorarlberg accounting for more than 50% of the population of Vorarlberg at that time. Enrollment in the HS was voluntary, and costs were covered by Austrian health insurance. All subjects participated solely in the context of medical prevention and did not see their physician for any signs or symptoms of cardiovascular or other diseases.

At OS, all participants were patients in the cardiology unit of the tertiary care hospital in Feldkirch (single center) who were consecutively referred to angiography and/or sonography for the evaluation of suspected or established ASCVD. Only patients who had participated in the HS were enrolled in the OS, and those with acute coronary syndrome were excluded.

This study thus contains (i) data at HS of 1497 subjects assumed to be in a healthy condition, (ii) data at OS of the same subjects when they were 15 years older, by then suspected to have ASCVD and being at a high cardiovascular event risk, and (iii) follow-up data for another 21 years, altogether covering a time span of up to 36 years (Figure S1).

The present study conforms to the ethical guidelines of the 1975 Declaration of Helsinki and has been approved by the Ethics Committee of Vorarlberg, Austria, and the University of Innsbruck, Austria (EK-2-22013/0008, EK-Nr. 2006-6/2, and EK-2-18/2021-9). Written informed consent was given by all participants. The authors affirm that all participants provided informed consent for publication of anonymized data.

### Method detail

#### Clinical and laboratory analyses and study endpoints

Detailed descriptions of basic clinical data assessment are given elsewhere.^32^^,^^34^ Systolic and diastolic BP were measured by health professionals using the Riva–Rocci method under resting conditions in a sitting position on the day of hospital entry not later than 5 h after admission (OS) or during the general examination (HS). No self-reported measurements were included. BP measurements ranged from 240 to 80 mmHg for systolic and from 145 to 40 mmHg for diastolic BP. Patients with BP outside this range were excluded. Systolic and diastolic BP data were available for all 1497 patients at HS and OS, except for two missing values of diastolic BP at OS. BP categorization is described in the following.

Follow-up data were available for 1487 out of 1497 patients, amounting to a follow-up rate of >99%. The primary study endpoint was cardiovascular mortality, and the secondary endpoint was overall mortality.

#### BP categorization according to ACC/AHA and ESC/ESH

According to recent ACC/AHA high BP guidelines,^14^ patients were stratified into four categories (normal BP [systolic <120 and diastolic <80], elevated BP [systolic 120–129 and diastolic <80], hypertension stage 1 [systolic 130–139 or diastolic 80–89], and hypertension stage 2 [systolic ≥140 or diastolic ≥90]. Recent ESC/ESH guidelines^15^ are slightly different in particular regarding cut-offs for diastolic BP readings and definition of hypertension and suggest discriminating between optimal and normal BP [summarized: systolic ≤129 and diastolic ≤84], high normal BP [systolic 130–139 or diastolic 85–89], grade 1 hypertension [systolic 140–159 or diastolic 90–99], grade 2 hypertension [systolic 160–179 or diastolic 100–109], and grade 3 hypertension [systolic ≥180 or diastolic ≥110]), resulting in five categories (Figure S2).

### Quantification and statistical analysis

Differences between paired samples, as for baseline characteristics of the HS and the OS, were tested for statistical significance with the paired McNemar test for categorical variables and the paired T-test and the Wilcoxon test for continuous variables. Correlation analyses were performed by calculating Pearson’s test. For prognostic endpoints, adjusted hazard ratios (HRs) for the incidence of overall mortality or cardiovascular mortality were derived from Cox proportional hazards models. The proportional hazard assumption was checked by examination of scaled Schoenfeld residuals. Cox regression models were adjusted for age, the time between HS and OS (Δtime), sex, BMI, low-density lipoprotein (LDL) -cholesterol, high-density lipoprotein (HDL) -cholesterol, lipoprotein (LP) a, smoking status, antihypertensive treatment (beta blocker, calcium channel blocker, ACE inhibitors or AT2 antagonists), and type 2 diabetes status.

The number of missing values was negligible, and all were missing completely at random (MCAR) according to Little’s MCAR test, thus all data were analyzed according to complete case analysis.

To examine the potential utility of predictive biomarkers, the areas under the curve (AUC) of receiver operating characteristic (ROC) curves of composed models were compared by applying DeLong’s test or by calculating Harrell's C and Somers' D for time-dependent ROC curves. The net reclassification improvement (NRI) and the integrated discrimination improvement (IDI) indices were computed using the improveProb-function of the HMISC package in R.

*A priori* sample size and power calculation revealed that the study was sufficiently powered. The sample size and power calculation were conducted using G∗Power 3 software version 3.1.9.7.^35^ *A priori* sample size calculation showed that assuming a particularly small effect size of 0.1 when comparing differences between two paired groups, with a desired alpha-fault of 5% and a power of 80% or 95%, the total sample size should be at least 620 or 1084 respectively. A post-hoc power calculation revealed that our study comprising 284 patients suffering cardiovascular death and 1203 patients not suffering cardiovascular death, given an alpha-fault of 5% and an effect size of 0.37 (based on systolic BP at HS) was sufficiently powered (>99%) to reject the null hypothesis that BP is not different between those two groups.

All statistical analyses were performed with SPSS 28.0 for Windows and R statistical software v. 4.3.1 (key resources table).
