# Supplementary material for: The value of earlier-in-life systolic and diastolic blood pressure for cardiovascular risk prediction
Source: iScience. 2024 Feb 2;27(3):109097. doi: 10.1016/j.isci.2024.109097 (PMC10879708; doi:10.1016/j.isci.2024.109097)
Supplement: Document S1. Figures S1–S8 and Tables S1–S4 [file mmc1.pdf]

iScience, Volume *27*

Supplemental information

The value of earlier-in-life systolic and diastolic blood pressure for cardiovascular risk prediction

Andreas Leiherer, Wolfgang Brozek, Axel Muendlein, Hanno Ulmer, Christoph H. Saely, Peter Fraunberger, Gabriele Nagel, Emanuel Zitt, Heinz Drexel, and Hans Concin

Supplementary Figures

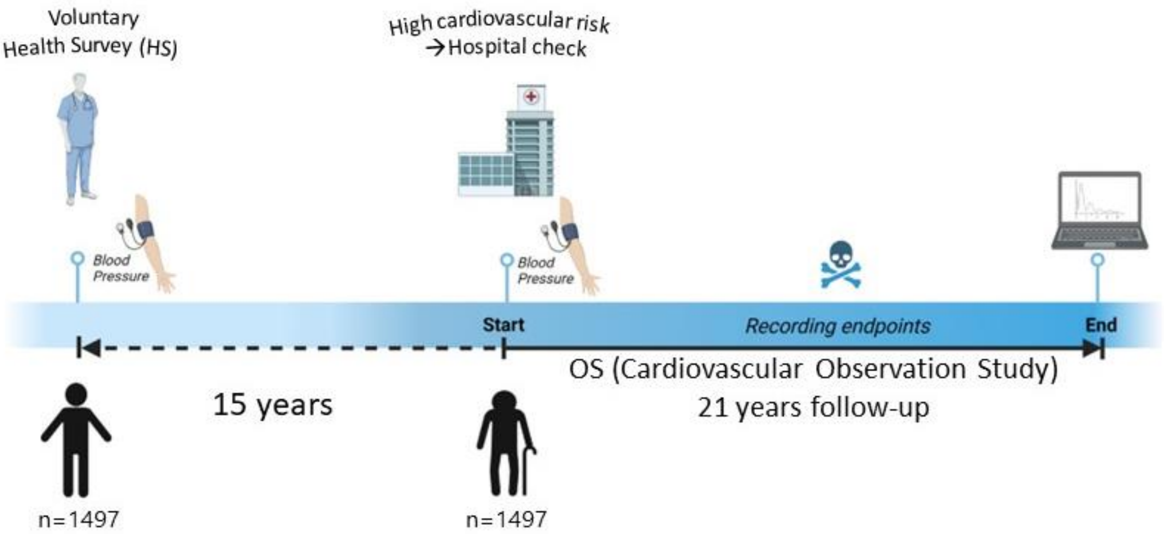

Supplementary Figure 1: Paired study design and timeline, related to STAR Methods. The first study was a large health survey (HS) of clinically healthy individuals whereas the second one was a more recent prospective cardiovascular observation study (OS) - initiated 15 years later on the identical subjects who were examined in hospital and included in the OS due to their high cardiovascular risk.

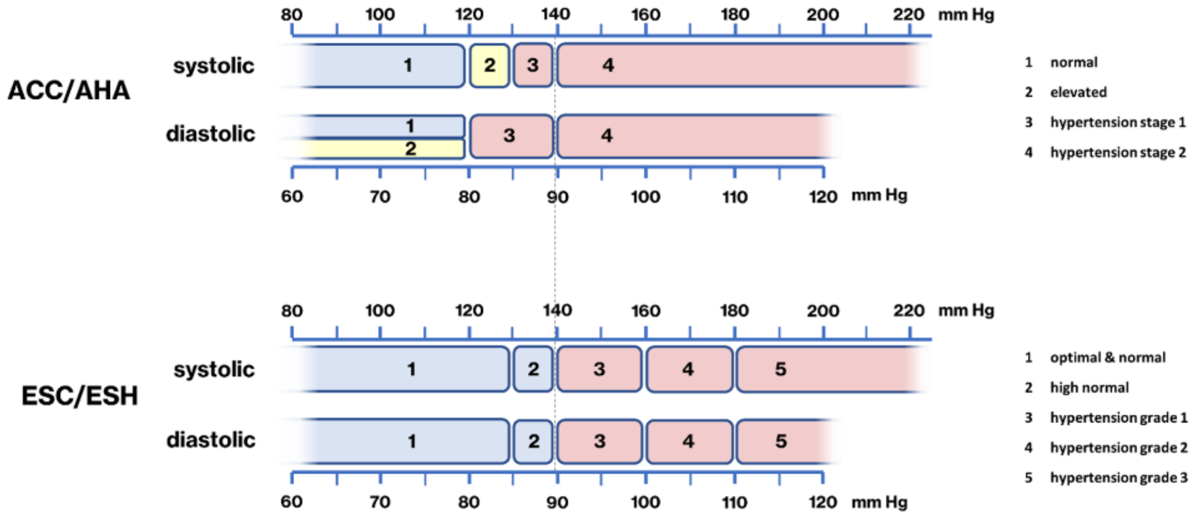

Supplementary Figure 2: BP categorization, related to STAR Methods. BP categories were built using systolic and diastolic measurements according to ACC/AHA guidelines and ESC/ESH guidelines, which are applying different cut-offs. The approximate crossing point between OS and HS-derived curves in Figure 1 is indicated by a dashed line.

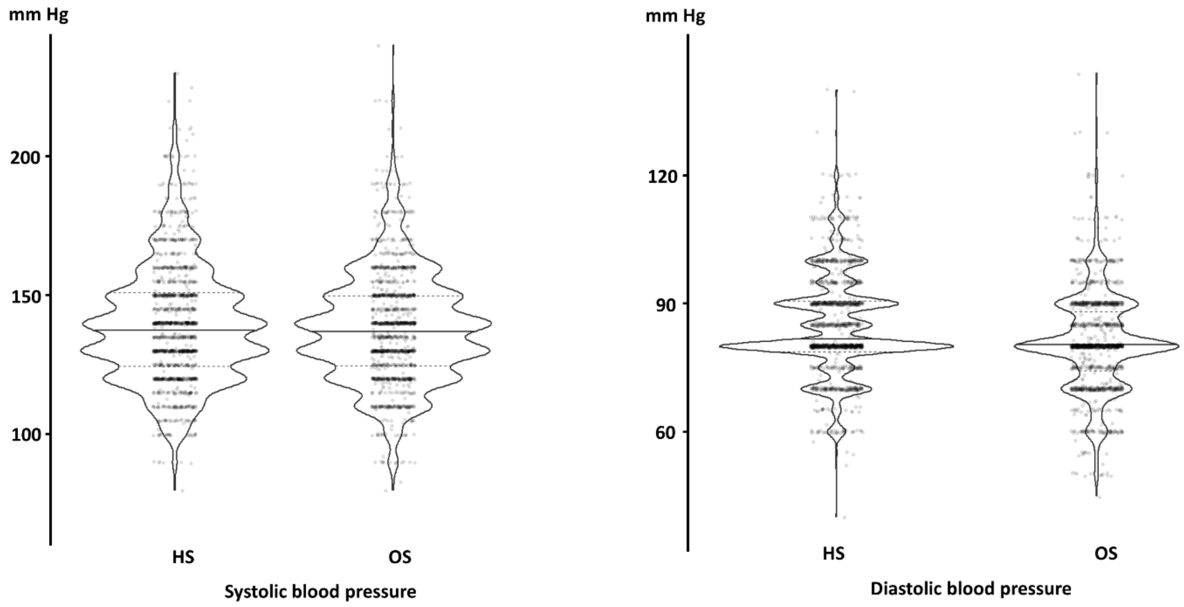

Supplementary Figure 3: Frequency of systolic and diastolic BP measurements at HS and OS, related to Table 1. The frequency of systolic (left panel) and diastolic (right panel) BP data measured at the health survey (HS) and 15 years later at the baseline of the observation study (OS) is represented by a violin plot and an overlay dot plot. The median is represented by a solid line and the 25th and 75th quartiles by dashed lines.

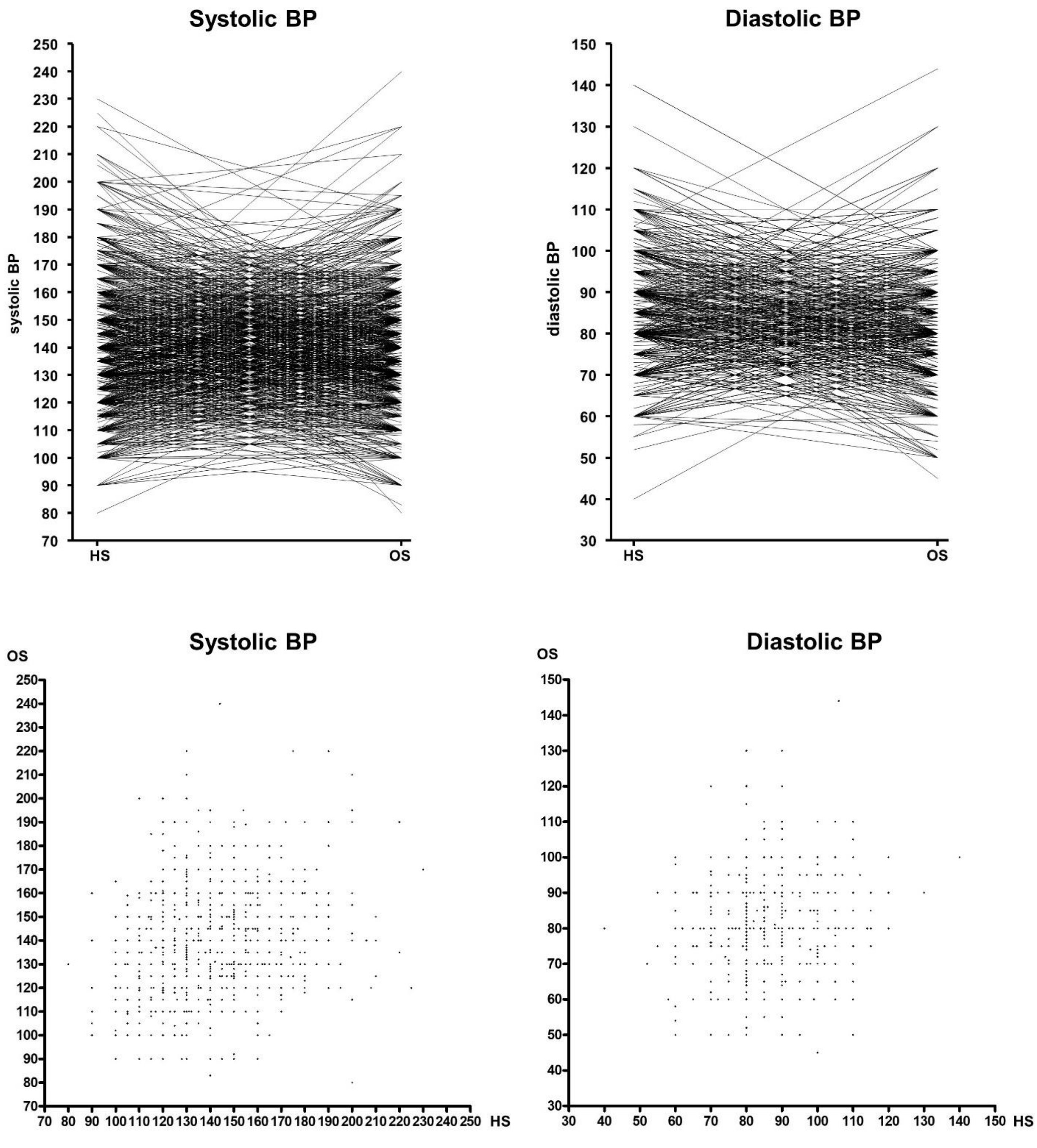

Supplementary Figure 4: Relation of blood pressure at the health survey and at the cardiovascular observation study, related to Table 1. The figure illustrates the systolic (left) and diastolic (right) BP of each individual subject at HS and OS as a paired plot with connecting lines (above) and as a scatter plot (below).

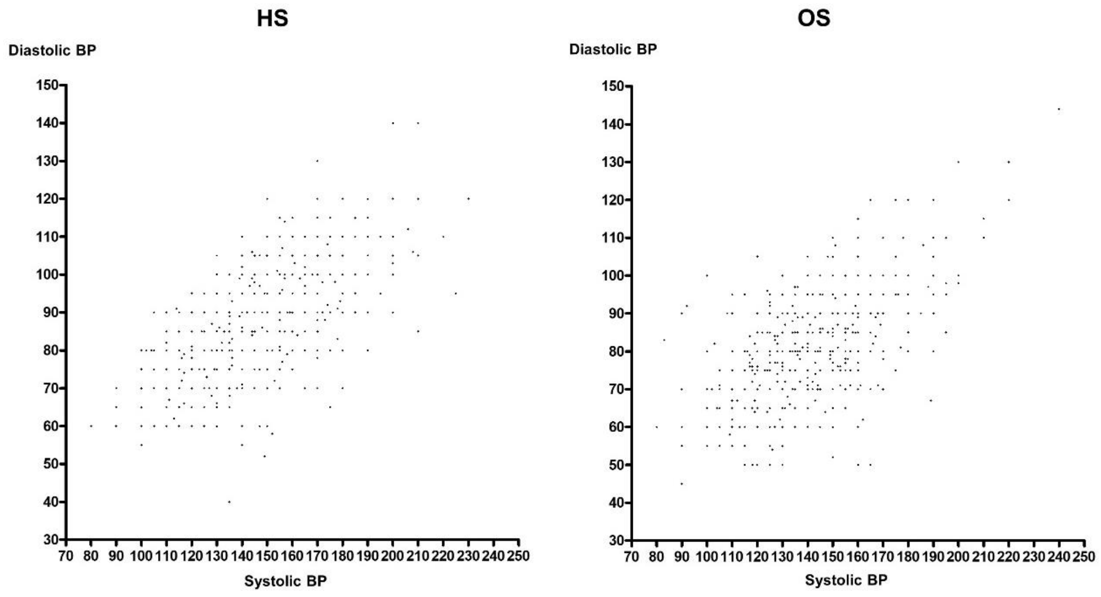

Supplementary Figure 5: Correlation of systolic and diastolic BP, related to STAR Methods. The scatter plot illustrates the systolic and diastolic BP of each individual subject at HS (left) and OS (right).

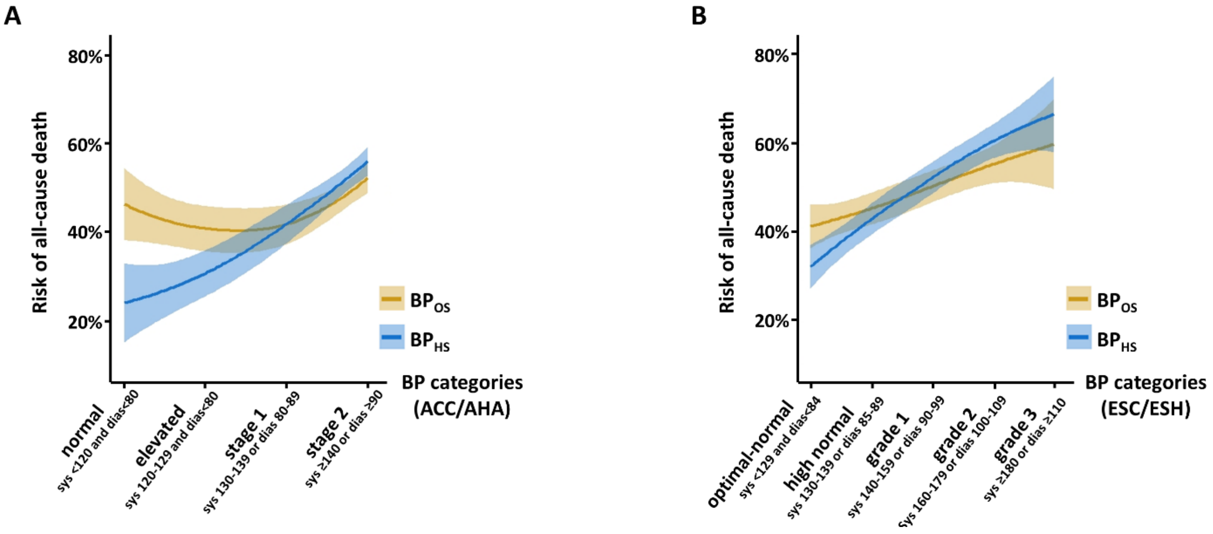

Supplementary Figure 6: Risk curves for overall mortality with blood pressure categories measured at the health survey and at the cardiovascular observation study, related to Figure 1. Curves are reflecting the risk of overall mortality during the follow-up of the cardiovascular observation study (OS) associated with blood pressure at OS or at the health survey (HS). Curves are calculated according to loess (LOcally WEighted Scatter-plot Smoother) with 95% confidence intervals. BP categories were built using systolic and diastolic measurements according to ACC/AHA guidelines (A) and ESC/ESH guidelines (B), respectively.

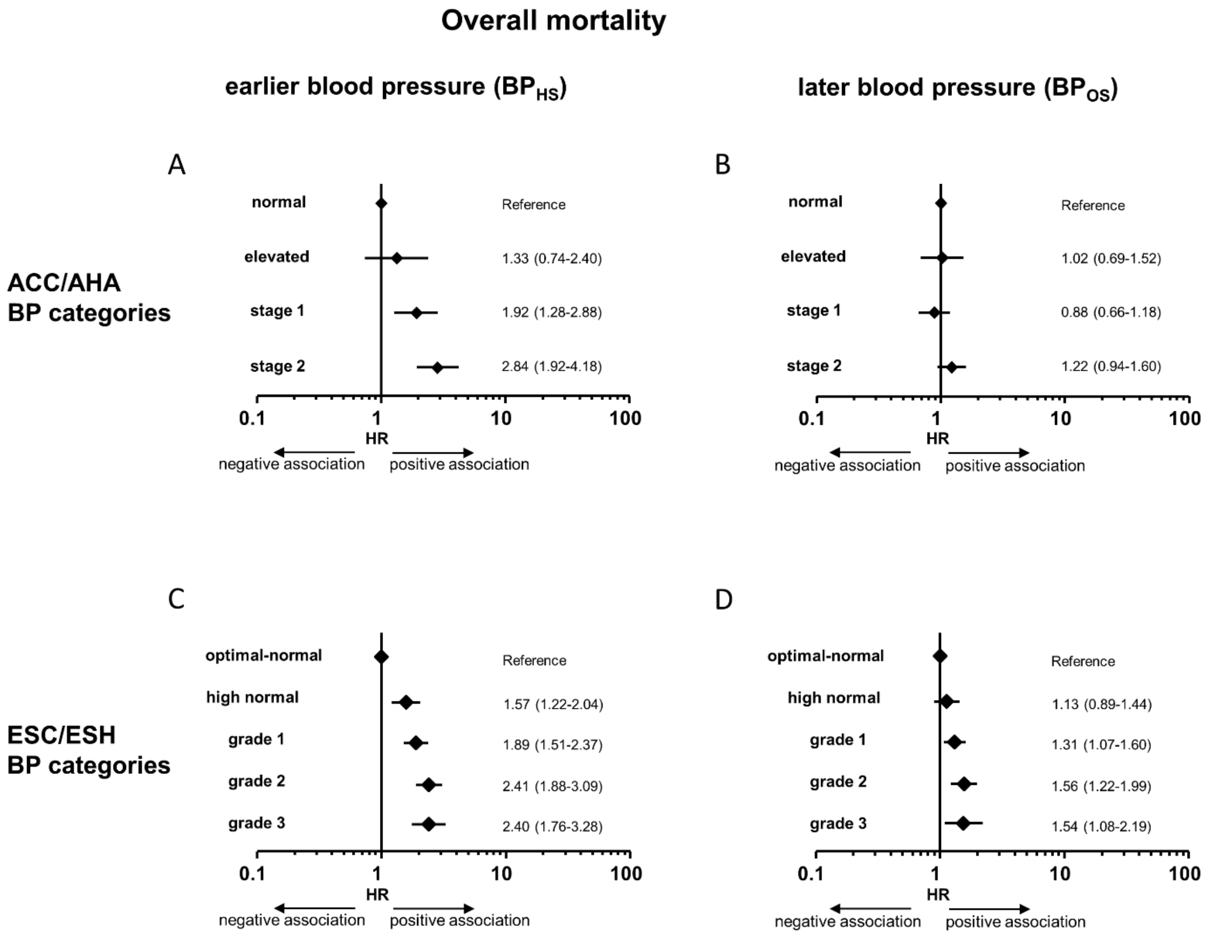

Supplementary Figure 7: Association of blood pressure categories assessed at the health survey and at the baseline of the cardiovascular observation study with overall mortality, related to Figure 2. Forest plots represent hazard ratios with 95% confidence interval of Cox regression analyses for the association between all-cause death and BP categories assessed at the health survey (HS; A+C) or at the observation study (OS; B+D). Categories were built according to ACC/AHA (A+B) or ESH/ESC guidelines (C+D).

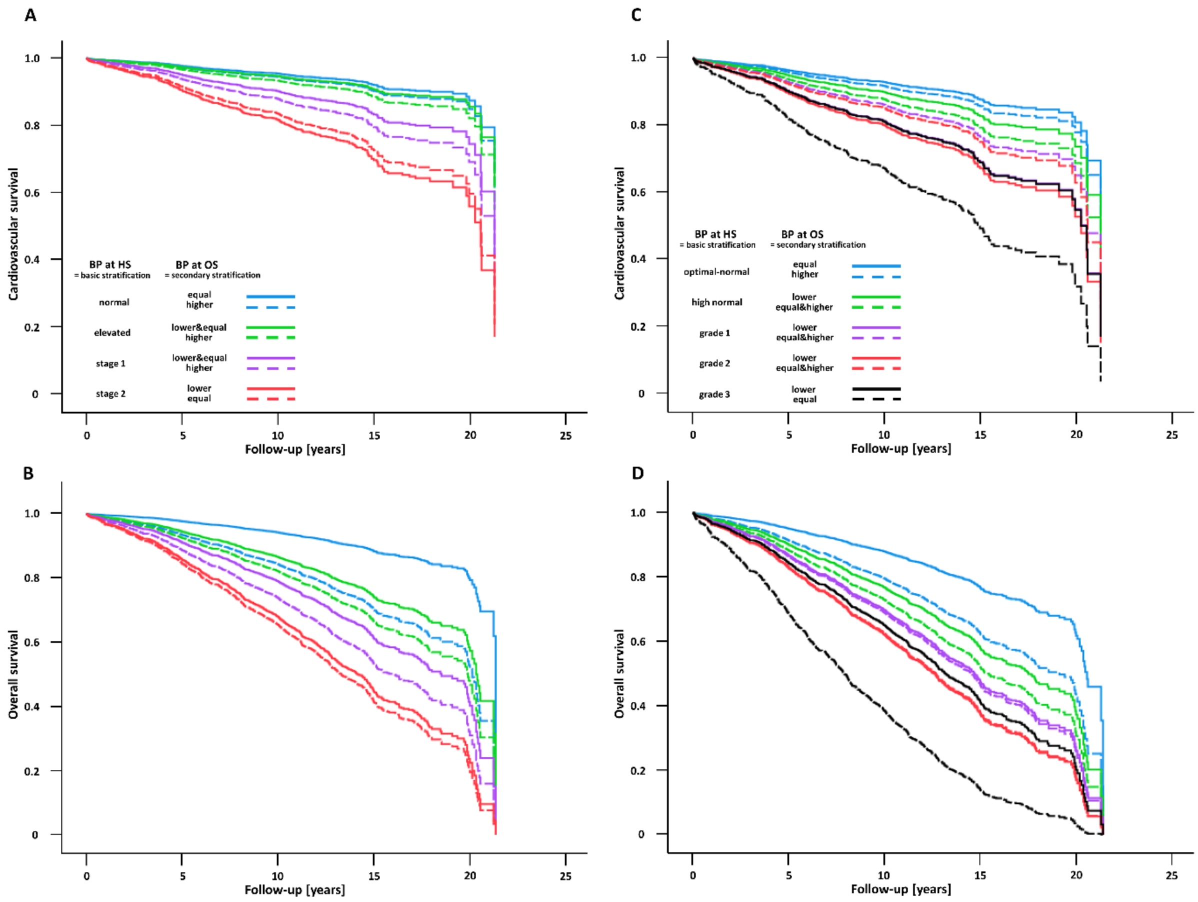

Supplementary Figure 8: Survival across blood pressure categories according to a basic and a secondary stratification, related to Figure 2. The plot represents adjusted Cox survival curves indicating cardiovascular (A+C) and overall survival (B+D) according to BP at HS (basic stratification: categories (colors)) and at OS (secondary stratification: two categories (line types)). The basic stratification uses BP measurements at HS according to ACC/AHA (four categories, A+B) or according to ESC/ESH (five categories, C+D). The secondary stratification uses the BP trend between HS and OS.

Supplementary Tables

Categorization	Outcome	BP at	model	HR [95%CI]	P-value
ACC/AHA	Cardiovascular mortality	HS	1	1.57 [1.32-1.86]	< 0.001
			2	1.36 [1.11-1.66]	0.003
			3	1.31 [1.07-1.61]	0.009
		OS	1	0.97 [0.86-1.10]	0.653
			2	0.90 [0.79-1.03]	0.119
			3	0.88 [0.77-1.00]	0.057
	Overall mortality	HS	1	1.44 [1.30-1.60]	<0.001
			2	1.17 [1.04-1.31]	0.007
			3	1.18 [1.06-1.33]	0.004
		OS	1	1.11 [1.02-1.20]	0.017
			2	1.00 [0.91-1.09]	0.995
			3	1.00 [0.91-1.09]	0.922
ESC/ESH	Cardiovascular mortality	HS	1	1.30 [1.18-1.43]	< 0.001
			2	1.16 [1.04-1.29]	0.009
			3	1.13 [1.01-1.26]	0.034
		OS	1	1.05 [0.94-1.16]	0.386
			2	1.00 [0.90-1.12]	0.935
			3	0.98 [0.88-1.10]	0.716
	Overall mortality	HS	1	1.26 [1.19-1.34]	<0.001
			2	1.11 [1.04-1.19]	0.003
			3	1.12 [1.04-1.20]	0.002
		OS	1	1.14 [1.07-1.22]	<0.001
			2	1.07 [1.00-1.15]	0.059
			3	1.06 [0.99-1.14]	0.113

Supplementary Table 1: Association of BP categories with overall mortality and cardiovascular mortality, related to Figure 2. BP categories were assessed either at the HS or 15 years later, at the OS and were built according to ACC/AHA guidelines and ESC/ESH guidelines, respectively. Hazard ratios (HR) denote the risk associated with an increase by one BP step category. Model 1 represents an unadjusted model approach including all patients. Model 2 is based on model 1 comprising variables age, Δage (if applicable), sex, BMI, LDL-cholesterol, HDL-cholesterol, and LP(a). Model 3 comprises all variables of model 2 and in addition beta blocker treatment and ACE inhibitor, AT2 antagonist, or Calcium channel blocker treatment, furthermore the diabetes status, and ever-smoking status.

Outcome	BP at		HR [95%CI]	P-value
Cardiovascular mortality	HS	systolic<br>diastolic	1.17 [1.11-1.23]<br>1.20 [1.09-1.32]	<0.001<br><0.001
	OS	systolic<br>diastolic	1.04 [0.98-1.10]<br>0.82 [0.73-0.92]	0.213<br><0.001
Overall mortality	HS	systolic<br>diastolic	1.15 [1.12-1.19]<br>1.17 [1.10-1.24]	<0.001<br><0.001
	OS	systolic<br>diastolic	1.08 [1.04-1.12]<br>0.90 [0.84-0.97]	<0.001<br>0.005

Supplementary Table 2: Association of systolic and diastolic BP with cardiovascular mortality and overall mortality, related to Figure 3. Systolic and diastolic BP was measured either in the course of the HS or 15 years later, in the course of the OS. Hazard ratios (HR) were calculated for each 10 mm Hg increase in BP.

Categori-zation	Outcome	Basic stratification (BP at HS)	Sec. stratification (trend HS→OS)	HR [95%CI]	P-value
ACC/AHA	Cardiovascular mortality	normal	equal	1	-
		normal	higher	1.25 [0.26-6.01]	<0.001
		elevated	lower&equal	1.16 [0.11-12.85]	0.901
		elevated	higher	1.51 [0.29-7.80]	0.621
		stage 1	lower&equal	2.21[0.53-9.27]	0.278
		stage 1	higher	2.81 [0.68-11.63]	0.154
		stage 2	lower	4.33 [1.06-17.61]	0.041
		stage 2	equal	3.84 [0.95-15.55]	0.059
	Overall mortality	normal	equal	1	-
		normal	higher	2.93 [0.88-9.72]	0.079
		elevated	lower&equal	2.41 [0.49-11.92]	0.282
		elevated	higher	3.44 [1.00-11.80]	0.050
		stage 1	lower&equal	3.99 [1.26-12.65]	0.019
		stage 1	higher	5.22 [1.66-16.43]	0.005
		stage 2	lower	6.46 [2.06-20.24]	0.001
		stage 2	equal	7.06 [2.26-22.01]	<0.001
ESH/ESC	Cardiovascular mortality	optimal-normal	equal	1	-
		optimal-normal	higher	1.20 [0.63-2.30]	0.573
		high normal	lower	1.43 [0.65-3.16]	0.371
		high normal	equal&higher	1.81 [0.97-3.37]	0.064
		grade 1	lower	2.82 [1.57-5.08]	<0.001
		grade 1	equal&higher	2.03 [1.13-3.65]	0.018
		grade 2	lower	2.99 [1.64-5.42]	<0.001
		grade 2	equal&higher	2.18 [1.01-4.71]	0.048
		grade 3	lower	2.77 [1.42-5.42]	0.003
		grade 3	equal	5.36 [1.76-16.30]	0.003
	Overall mortality	optimal-normal	equal	1	-
		optimal-normal	higher	1.83 [1.19-2.82]	0.006
		high normal	lower	2.05 [1.23-3.40]	0.006
		high normal	equal&higher	2.52 [1.65-3.85]	<0.001
		grade 1	lower	2.81 [1.85-4.26]	<0.001
		grade 1	equal&higher	2.89 [1.93-4.33]	<0.001
		grade 2	lower	3.64 [2.41-5.51]	<0.001
		grade 2	equal&higher	3.63 [2.23-5.89]	<0.001
		grade 3	lower	3.23 [2.04-5.14]	<0.001
		grade 3	equal	7.35 [3.57-15.14]	<0.001

Supplementary Table 3: Cardiovascular and overall survival across blood pressure categories according to a basic and a secondary stratification, related to Figure 2 and supplementary Figure 8. The table summarizes HRs of BP categories according to a basic and a secondary stratification. The basic stratification uses BP measurements at HS according to ACC/AHA (four categories) or according to ESC/ESH (five categories). The secondary stratification uses the BP trend between HS and OS.

**Prediction of cardiovascular mortality**	**AUC**	**DeLong's Z (P-value)**	**Harrell's C**	**Somers' D**	**IDI (P-value)**	**NRI (P-value)**
BP_{HS} cat-ACC/AHA [a]	0.585		0.588	0.176		
BP_{OS} cat-ACC/AHA [b]	0.496‡	4.019 [a vs.b] (<0.001)	0.496	-0.008	0.0385 [a vs.b] (<0.001)	0.304 [a vs. b] (<0.001)
BP_{HS} cat-ESC/ESH [c]	0.594	1.266 [c vs. a] (0.205)	0.592	0.183	0.0031 [c vs. a] (0.502)	0.038 [c vs. a] (0.563)
BP_{OS} cat-ESC/ESH [d]	0.509	3.589 [c vs. d] (<0.001)<br>1.667 [b vs. d] (0.096)	0.512	0.023	0.0339 [c vs.d] (<0.001)<br>7.79 e-5 [b vs. d] (0.965)	0.292 [c vs. d] (<0.001)<br>0.015 [b vs. d] (0.817)
Systolic BP_{HS} [e]	0.606		0.605	0.210		
Systolic BP_{OS} [f]	0.521	3.509 [e vs. f] (<0.001)	0.526	0.051	0.0393 [e vs. f] (<0.001)	0.302 [e vs. f] (<0.001)
Diastolic BP_{HS} [g]	0.574	1.837 [e vs.g] (0.066)	0.570	0.141	0.0232 [e vs.g] (<0.001)	0.268 [e vs.g] (<0.001)
Diastolic BP_{OS} [h]	0.436‡	4.782 [f vs.h] (<0.001)<br>5.427 [g vs. h] (<0.001)	0.427	-0.145	0.0152 [f vs.h] (0.020)<br>0.0004 [g vs. h] (0.958)	0.100 [f vs.h] (0.130)<br>0.007 [g vs. h] (0.919)
**Prediction of overall mortality**	**AUC**	**DeLong's Z (P-value)**	**Harrell's C**	**Somers' D**	**IDI (P-value)**	**NRI (P-value)**
BP_{HS} cat-ACC/AHA [a2]	0.602		0.572	0.145		
BP_{OS} cat-ACC/AHA [b2]	0.544	3.297 [a2 vs. b2] (0.001)	0.520	0.041	0.0377 [a2 vs. b2] (<0.001)	0.334 [a2 vs. b2] (<0.001)
BP_{HS} cat-ESC/ESH [c2]	0.622	3.141 [c2 vs. a2] (0.002)	0.583	0.167	0.0067 [c2 vs. a2] (0.040)	0.069 [c2 vs. a2] (0.167)
BP_{OS} cat-ESC/ESH [d2]	0.560	3.360 [c2 vs. d2] (0.001)<br>2.481 [d2 vs. b2] (0.013)	0.533	0.066	0.0255 [c2 vs.d2] (<0.001)<br>0.0054 [d2 vs. b2] (<0.001)	0.264 [c2 vs. d2] (<0.001)<br>0.105 [d2 vs. b2] (0.026)
Systolic BP_{HS} [e2]	0.645		0.600	0.199		
Systolic BP_{OS} [f2]	0.570	4.065 [e2 vs. f2] (<0.001)	0.542	0.084	0.0301 [e2 vs. f2] (<0.001)	0.361 [e2 vs. f2] (<0.001)
Diastolic BP_{HS} [g2]	0.589	4.398 [e2 vs. g2] (<0.001)	0.558	0.116	0.0243 [e2 vs.g2] (<0.001)	0.417 [e2 vs.g2] (<0.001)
Diastolic BP_{OS} [h2]	0.456‡	7.766 [f2 vs.h2] (<0.001)<br>6.725 [g2 vs. h2] (<0.001)	0.457	-0.087	0.0042 [f2 vs.h2] (0.204)<br>0.0096 [g2 vs. h2] (0.002)	0.101 [f2 vs.h2] (0.054)<br>0.093 [g2 vs. h2] (0.073)

Supplementary Table 4: Predictive performance measures of BP on cardiovascular mortality and overall mortality, related to STAR Methods. The table summarizes AUC, Harrell´s C and Somers´ D as well as Z of DeLong's test, the integrated discrimination improvement (IDI), and the net reclassification index (NRI) of different single predictors (BP) of BP measured at HS (BP_{HS}) or OS (BP_{OS}). An inverse relation between diastolic BP and endpoints is reflected by AUC <0.5 (‡). P-values are given for two-tailed tests, if applicable.
